# Intelligence

**Published:** 1809-12

**Authors:** 


### INTELLIGENCE.

We wished very much to procure an accurate detail of all the symptoms in the man who was bitten by the rattle snake. All we have hitherto learned is, that mortification took place in several phalanges of the fingers, and that separation had begun before death. Another, and still more important event, of which we have received many particulars, and to the dissections of some of which we were present, is the death of three horses under symptoms of hydrophobia. It is ascertained that two of them were bitten by the same dog, and there is reason to believe the same of the third. The great importance of every part of the question, makes us anxious to be not less minute than accurate in our whole information, which we trust will prove our excuse for delaying the account of this as well as the other event till our next Number.

We have frequently had occasion to notice the progress made on the Continent, in extracting sugar from the beet-root; and it now appears, that the yellow beet, when sliced and kiln-dried, furnishes an excellent substitute for coffee, particularly if ground with a small quantity of Turkey or West India coffee. It requires much less sugar than the foreign coffee, and is said to be much stronger. M. Vennen, of Coblentz, claims the merit of having discovered this new application of beet-root. He cautions those, by whom it is cultivated, against stripping the plant of its leaves for feeding cattle, as is generally practised, as it not only injures the growth of the plant, but materially alters the qualities of the juice.

Great exertions are making in every department of France to produce substitutes for sugar, and prizes are daily offered by the various economical societies of the continent, for the discovery of the most proper material for that purpose. The saccharine matter of grape has been the chief subject of the recent experiments of the French chemists.

M. Peron

M. Peron, during his voyage to the South Seas, collected a great number of that remarkable genus of animals, to which Linnæus gave the name of *Medusa*, and has increased that family to more than 150 species. In an account lately presented by him to the National Institute, their singularities are well expressed in the following terms:—"Their substance seems to be merely a coagulated water, yet the most important functions of life are exercised in it. Their multiplication is prodigious, yet we know nothing of the peculiar mode in which it is effected. They are capable of attaining several feet in diameter, and the weight of fifty or sixty pounds, yet their nutritive system escapes our eyes. They execute the most rapid and long continued movements, yet the details of their muscular system are imperceptible. They have a very active species of respiration, the true seat of which is a mystery. They appear extremely feeble, yet fish of considerable size form their daily prey, and dissolve in a few moments in their stomach. Many species of them shine in the night like balls of fire, and some sting or benumb the hand that touches them; yet the principles and agents of both these properties remain to be discovered. All the medusas have a gelatinous body, nearly resembling the cap of a mushroom, and hence denominated *umbrella*; but they differ in wanting, or having a mouth; in the mouth being simple, or multiplicious; in the presence or absence of a production, resembling a pedicle; and in the edges of this pedicle, or of the mouth itself, being furnished with *tenta cula*, or filaments, more or less numerous. From these characters M. Peron forms divisions and subdivisions, under which every possible kind of medusa may be arranged. Some of these animals exhibit beautiful colours.

In the evening of the 26th of June, a terrestrial water-spout appeared, about a league south-east of Carmagnole, in the department of the Po. The weather was stormy. The cloud, which gave rise to this meteor, was greyish, and not very large; but it began to lengthen on one side, forming as it were a very sharp tail, which approached the earth in a serpentine line. The cloud had then the shape of a reversed cone, part of which emitted a very perceptible yellowish light; this cone, about half way between the summit and base, might be eight or nine yards in circumference. As it approached the earth, a kind of cloud that looked like smoke, having also the appearance of a kind of cone, was formed, and its summit advanced towards the water-spout. The duration of this meteor was twenty minutes, during which it traversed a space of more than eight hundred yards, and then descended in a deluge of water. In its way, it overthrew a young oak, and stripped the bark from a mulberry tree, the roots of which were almost entirely laid bare, by the removal of the earth which covered them. The bark was reduced to a dry, whitish, and almost friable substance. The lower cone also exerted its fury upon the dust, which it raised, and the corn which was then cut in the fields, and which it carried away and dispersed. A man who who was in the line traversed by this phenomenon, feeling himself beginning to rise, held by a bush that he might not be carried away. A quarter of an hour after the disappearance of the water spout, there was a thunder storm with hail. The thermometer was at 18°, and the mercury in the barometer, which at first stood at twenty-seven inches six lines, rapidly fell 2 ½ lines.

Another phenomenon, attended however with still more mischievous effects, occurred on the 8th of July, near Aix, in the department of Mont Blanc. The wind was south, and the thermometer at 22°; the cloud in which it originated, appeared in the form of a water-spout, about six miles from Aix, at a considerable elevation. It proceeded along the chain of the Lesser Alps, situated north-west of Chamberry; it was slightly charged with electric matter, and carried along with it a prodigious mass of flakes of ice, with a tremendous noise. Having traversed the distance of about eighteen miles, along the summit of the mountains, a contrary current of wind meeting it above lake Bourget, about six miles from Aix, detached a portion which was carried toward the north-north-east; while the other continued its course westward, towards the Lyonnois. In both directions, the storm spread devastation through the vallies. The town of Annecy has not a single pane of glass or tile left whole. The lumps of ice were as large as a man's fist, some weighing three, three and a half, and even four pounds Numbers of the country people are wounded; several shepherds are killed, and great numbers of cattle killed and wounded. The desolation is general throughout a tract of forty-two miles. The progress of the column of ice along the mountain, opposite to Aix, exhibited the most terrific and at the same time imposing spectacle that can possibly be conceived.

After the extraordinary tide observed on the 4th of July last, in the whole gulf of Spezzia, and which is called by the Italians, *terremoto di mare*, (sea earthquake) the whole course of the tides there was totally deranged for the seven or eight succeeding days. The ebb and flood were sensibly perceived at intervals of a quarter of an hour, half an hour, and an hour, during that whole space of time. A sloop within sight of Leghorn, was overtaken the same day by this tide, which in a moment rendered the sea, before quite calm, extremely rough. The cause of this phenomenon is supposed to have some connection with the earthquakes, which the western part of Italy has so frequently experienced for some years, fresh shocks of which were felt by the towns and vallies of Suze and Pignerol on the 2d and 3d of July last. It cannot be doubted that earthquakes and volcanic eruptions frequently convulse the bottom of the sea, without occasioning the mischief which they produce on land, and without its being possible for them to be observed, even by navigators. The Italian expression for this phenomenon would therefore be perfectly correct, and founded upon the laws and effects of Nature.

Dr. Ramsbotham will commence his Lectures on the Science and Practice of Midwifery, on Wednesday January the 3d, 1810, at his House, No. 9, Old Jewry.

Dr. Clough, Physician, Manmidwife to the St. Mary-le-bone General Dispensary, &c. will, on Monday, the 10th of January, at Ten in the Morning, commence his Spring Course of Lectures on Midwifery, including the Diseases of Women and Children, at his House, 68, Berners Street; and for the convenience of Students, attending other Classes and Hospitals) his Evening Course on Tuesday the 9th, at Seven o'Clock.—For a Syllabus and Prospectus of the Course, apply to Dr. Clough as above.

Dr. Adams's edition of Mr. Hunter will be published by the middle of the current month.

In the course of this month will be published Dr. Edward Goodman Clarke's new work, entitled, Pharmacopœiarum Collegiorum Regalium Medicorum, Londini, Edinburgi, nec non Eblanæ Conspectus Medicus, Virtutes, Doses et Morbos quibus adhibentur Medicamenta, exhibens.

Dr. Stancliffe, well known as a popular lecturer, is about to publish a volume of Chemical Experiments, for the use of Students, consisting of nearly one thousand, in the various branches of the science.

Dr. Stokes has in considerable forwardness, a Botanical Materia Medica, consisting of the generic and specific characters of the plants used in medicine and diet, with symptoms and references to medical authors.

